# A Detailed Study of Infection Following Custom-Made Porous Hydroxyapatite Cranioplasty: Risk Factors and How to Possibly Avoid Device Explantation

**DOI:** 10.3390/jcm14186443

**Published:** 2025-09-12

**Authors:** Francesca Carolina Mannella, Francesca Faedo, Johan Pallud, Salvatore Chibbaro, Marta Fumagalli, Giuseppe Danilo Norata, Ismail Zaed, Franco Servadei

**Affiliations:** 1 Department of Pharmacological and Biomolecular Sciences “Rodolfo Paoletti”, Università degli Studi di Milano, 20133 Milan, Italy; francesca.mannella@unimi.it (F.C.M.); marta.fumagalli@unimi.it (M.F.); danilo.norata@unimi.it (G.D.N.); 2 Department of Biomedical Sciences, Humanitas University, 20072 Milan, Italy; francesca.faedo@humanitas.it; 3 Neurosurgery Department, GHU Paris Psychiatry and Neurosciences, Sainte-Anne Hospital, 75014 Paris, France; johanpallud@hotmail.com; 4 Institute of Psychiatry and Neuroscience of Paris (IPNP), INSERM UMR 1266, Paris-Cité University, 75014 Paris, France; 5 Neurosurgery Unit, Department of Medical and Surgical Sciences and Neurosciences, Siena University Hospital, Azienda Ospedaliera-Universitaria, 53100 Siena, Italy; schibbaro@hotmail.com; 6 Department of Neurosurgery, Neurocenter of the Southern Switzerland, Regional Hospital of Lugano, Ente Ospedaliero Cantonale, 6900 Lugano, Switzerland; ismail.zaed@eoc.ch; 7 Global Neurosurgery Programme, Fondazione IRCCS Istituto Neurologico Besta, 20133 Milan, Italy

**Keywords:** cranioplasty, porous hydroxyapatite, postoperative infection, antibiotic treatment

## Abstract

**Background/Objectives:** Postoperative infection is a significant complication following cranioplasty procedures. This study aimed to assess infection risk factors and clinical outcomes in patients undergoing cranioplasty with custom-made porous hydroxyapatite (PHA) implants, with a particular focus on treatment strategies used to manage infections and avoid implant explantation. **Methods:** This retrospective multicenter analysis included 984 patients who underwent PHA cranioplasty as part of a post-market clinical follow-up. Clinical data included demographics, surgical characteristics, infection features, microbiological results, infection management strategies, and outcomes. Associations with infection risk and implant explantation were assessed using chi-square tests. **Results:** Seventy-six patients (7.7%) developed postoperative infections. Infection risk was significantly associated with second-line procedures (*p* = 0.011) and implant location (*p* = 0.037). Most infections were superficial (92.1%) and early-onset (≤2 months from the surgery, 61.9%), with *Staphylococcus* spp. as the predominant pathogens. Explantation occurred in 77.6% of infected cases. The infection management strategy—whether initial conservative treatment with antibiotics alone (n = 18 of which 11.1% explanted) or surgical reoperation (n = 58 of which 93.8% explanted)—along with surgical cleaning and local (in situ) antibiotic use alone, was significantly associated with explantation outcomes (all *p* < 0.001). Among 18 patients treated with systemic antibiotics alone, 88.9% retained their implants. Notably, all successful cases had received broad-spectrum antibiotics for at least 4 weeks. Local antibiotic therapy was administered in 13 patients; no explants occurred among those who also received prolonged systemic treatment. Pathogen type was not significantly associated with the risk of explantation. **Conclusions:** Prolonged systemic antibiotic therapy, especially when combined with local treatment, may allow implant retention in selected infections, supporting individualized, conservative management strategies.

## 1. Introduction

Cranioplasty is a commonly performed neurosurgical procedure aimed at restoring cranial protection, improving cerebral hemodynamics, and addressing cosmetic concerns following decompressive craniectomy [1,2]. Despite its benefits, it carries a relatively high risk of complications [3,4], with postoperative infection being among the most significant and challenging [5,6,7]. Reported infection rates vary widely—from 5% to 26%—and are associated with implant removal, delayed reconstruction, increased patient morbidity, and greater healthcare resource utilization [4].

The optimal management of post-cranioplasty infections remains a subject of ongoing debate [8]. While traditional strategies often involve implant explantation followed by delayed reimplantation, emerging evidence suggests that one-stage procedures or more conservative approaches may be feasible in selected cases [9]. However, treatment decisions are frequently influenced by institutional practices, surgeon preference, and the limited availability of robust, comparative multicenter data [6,10].

Porous hydroxyapatite (PHA), a calcium phosphate ceramic that mimics natural bone, is widely used for cranial reconstruction due to its regenerative potential. Its interconnected porous structure supports osteogenic cell migration, vascular ingrowth, and progressive osseointegration, while the custom-made 3D design ensures anatomical fit and favorable cosmetic outcome. However, the PHA implants are structurally fragile before osteointegration—typically achieved within six months—during which time protective measures such as helmets may be required. Despite these limitations due to its biological properties, PHA is increasingly adopted for complex cranial reconstructions in both adult and pediatric patients, and more than 9500 implants have been performed only in Europe [9].

To address the above-mentioned gap, we conducted a retrospective, multicenter European study aimed at characterizing current practices, outcomes, and predictive factors associated with the management of postoperative infections in cranioplasty with PHA. By analyzing data from multiple centers across Europe, this study seeks to provide a comprehensive overview of real-world strategies and outcomes, contributing valuable insights to inform clinical decision-making and guide future research.

## 2. Materials and Methods

### 2.1. Study Design and Patient Population

This retrospective, non-interventional, multicenter European study presents an analysis of the outcomes of cranioplasty using custom-made porous hydroxyapatite (PHA) implants (CustomBone Service, FinCeramica Faenza Spa, Faenza, Italy), with a specific focus on the management and treatment outcomes of postoperative infections. Only European centers using this particular cranioplasty material were included in the study (a list of centers and clinicians is provided in Supplemental Table S1). All cases of PHA reconstruction were performed using a direct preoperative 3D reconstruction based on the patient’s CT scan, in contrast to the technique of preoperative prototyping and intraoperative molding with PMMA recently reported [11]. The analysis is based on the same post-market clinical follow-up (PMCF) cohort previously described [12], which has since been updated and expanded to include a total of 984 patients (previously 687). While the previous publication was primarily descriptive of cranioplasty with PHA, focusing on infection rates in adult and pediatric populations, the present study offers a more comprehensive, multicentric evaluation of infection characteristics, management strategies, and explantation risk factors, incorporating newly defined treatment categories and additional statistical comparisons. The main objective of this study was to describe infection characteristics and their management to provide practical recommendations for improving treatment strategies following cranioplasty with PHA implants.

### 2.2. Data Collection

According to the Medical Devices Regulation (EU) 2017/745 (MDR), clinical follow-up of marketed medical devices is required to ensure continuous updating of the clinical evaluation process. Consequently, proactive post-market surveillance activities are regularly conducted to monitor the long-term safety of the implanted devices. This surveillance, initiated in December 2018, complies with the provisions of Annex X 1.1-quarter of Directive 93/42/EEC (as amended by Directive 2007/47/EC) and aligns with MEDDEV guidelines (2.7/1 rev.4 and 2.12/1 rev.8). Data were collected using a standardized clinical protocol and dedicated case report form, as described in Mannella et al. [12] (a copy of the protocol is provided in Supplemental File S1). All patients met the general criteria defined in the CustomBone Service instructions for use. No additional inclusion or exclusion criteria were applied. Relevant clinical data—such as demographic and epidemiological details, primary diagnosis, postoperative complications, infection characteristics and treatment, and explantation—were reviewed and validated by a contract research organization (CRO) collaborating with our institutions and the treating neurosurgeons (Supplemental Table S1).

### 2.3. Definitions and Classification Criteria

To ensure clarity and consistency in data interpretation, the following definitions and classifications were applied in this study:

Cranioplasty line of treatment

- First-line treatment: defined as the initial PHA cranioplasty performed after decompressive craniectomy.
- Second-line treatment: referred to cases in which a previous implant (made of a different material) had been removed due to complications and was subsequently replaced with a PHA prosthesis.

Implant location

Implant location was classified based on anatomical placement, including:

- Unilateral fronto-parieto-temporal: involving the frontal, parietal, and temporal bones on one side of the skull
- Bifrontal: involving both frontal bones
- Other: including parietal, occipital, or mixed regions not falling under the main categories.

Infection onset

- Early infections: defined as those occurring within 2 months after cranioplasty.
- Late infections: defined as those occurring more than 2 months after the procedure.

Infection Depth

Infections were classified according to internationally accepted criteria from the U.S. Centers for Disease Control and Prevention (CDC) and the National Healthcare Safety Network (NHSN) [13,14,15]. The classification was based on clinical documentation and treatment requirements recorded in the patients’ medical record, such as the following:

- Superficial infections: Limited to the skin and subcutaneous tissue at the surgical site.
- Deep infections: Involved deeper soft tissues (fascia, muscle layers, joint space, or bone) and required systemic antibiotic therapy and/or surgical debridement.

Infection Management Strategies

Infection management was categorized into six mutually exclusive strategies:

1. Antibiotic treatment only: A conservative approach in which systemic antibiotics were administered (intravenously and/or orally) without planned surgical intervention. Explantation was performed only in cases of pharmacological treatment failure.
2. Explantation without reimplantation: Permanent implant removal without subsequent replacement.
3. Reoperation with a different implant material: Replacement with a new implant of an alternative material, such as titanium or polyetheretherketone (PEEK).
4. Reoperation with a backup PHA implant: Reimplantation using a second identical PHA implant delivered at the time of the initial cranioplasty manufacturing and stored for up to one year for potential use in case of explantation.
5. Reoperation with a newly manufactured PHA implant: A new implant produced specifically for the patient after explantation.
6. Reoperation with the same PHA implant: The original implant was re-sterilized and reimplanted. In every case, reimplantation occurred at least a few months after explantation.

Other Associated Treatments

- Surgical debridement: removal of infected or necrotic tissue from the surgical site to reduce microbial load and promote healing. This procedure was typically performed in cases of severe or unresponsive infection, often in combination with systemic antibiotic therapy.
- In situ (local) antibiotic therapy: direct application of antimicrobial agents at the surgical site, generally in combination with systemic antibiotic treatment.

Prolonged Systemic Antibiotic Therapy

Defined as the systemic administration of antibiotics (intravenous and/or oral) for a minimum duration of 4 weeks. In this study cohort, prolonged treatments ranged from 4 to 12 weeks. All successfully treated cases received at least 4 weeks of therapy, while cases requiring early explantation typically received only 2 weeks.

### 2.4. Antibiotic Protocols

There was no universal antibiotic protocol applied across all patients. Each participating hospital followed its own internal clinical guidelines and antibiotic stewardship protocols, which varied based on local microbiological profiles, surgical practices, and institutional standards. Details of systemic and in situ antibiotic treatments are reported in Supplemental Table S2.

### 2.5. Statistical Analysis

Descriptive statistics and bivariate analyses using chi-square tests and exact tests were performed using SPSS Statistics version 29.0.2.0 (IBM Corp., Armonk, NY, USA, released 2021), in conjunction with information stored in the FinCeramica clinical database. Crude odds ratios and 95% CIs were calculated from 2 × 2 contingency tables using standard formulas. Due to the limited number of cases in several treatment subgroups, binary logistic regression was not conducted, as the small sample size precluded the development of a statistically reliable model. Although logistic regression was considered to adjust for potential confounders, the limited number of cases in certain treatment subgroups, combined with quasi-complete separation, prevented the development of a statistically reliable multivariable model. For this reason, only descriptive and unadjusted analyses are presented, and results should be interpreted as exploratory.

### 2.6. Ethical Considerations

Data collection was based exclusively on retrospective analysis of existing medical records and was conducted in accordance with the ethical principles of the 1964 Declaration of Helsinki and its subsequent amendments. The study also adhered to applicable European regulatory standards and Good Clinical Practice (GCP–ICH E6), including statistical guidelines outlined in ICH E9. Informed consent for the use of clinical data was obtained by the manufacturer from the surgeons at the time each implant was requested. As a result, no additional ethical approval was required from the participating centers involved in data collection. Prior to analysis, all data were aggregated and fully anonymized.

## 3. Results

### 3.1. Patient Demographics and Clinical Characteristics

A total of 984 patients who underwent custom-made porous hydroxyapatite (PHA) cranioplasty were included in the study. Table 1 summarizes the main epidemiological, clinical, and surgical characteristics of the cohort.

The median age at surgery was 41.5 years (IQR 25.0–54.0), and 64.3% of patients were male. The most frequent primary diagnoses leading to cranioplasty were trauma (n = 562, 57.1%), followed by vascular diseases (n = 205, 20.8%), tumor (n = 171, 17.4%), and skull malformation (n = 31, 3.2%). The majority of implants were placed in the fronto-parieto-temporal regions (n = 858, 87.2%). Overall, postoperative complications occurred in 13% (n = 128) of cases. The mean postoperative follow-up was 33.2 months (range: 1 month to 180 months).

### 3.2. Incidence and Risk Factors of Post-Cranioplasty Infections

Among the 984 patients, 76 (7.7%) developed a post-cranioplasty infection (Table 2).

Of these, 61.8% were male and 38.2% female. The median age in the infection group was 42.5 years (IQR 20.5–54.75). Nine patients (11.8%) were pediatric (2–13 years), while the remaining 67 (88.2%) were adults (≥14 years). Trauma was the most common primary diagnosis among infected patients (n = 43, 56.6%), followed by vascular diseases (n = 16, 21%), tumor (n = 11, 14.5%), malformation (n = 4, 5.3%), and other causes (n = 2, 2.6%). In most cases, cranioplasty had been performed as a first-line procedure (n = 53, 69.7%). Regarding implant location, the fronto-parieto-temporal region was involved in the majority of cases (n = 61, 80.3%), while bifrontal placement was reported in 10 patients (13.2%).

Chi-square tests were performed to assess epidemiological differences between patients who developed infections and the general cohort. Results showed no significant differences in sex, age, or primary diagnosis between patients with and without infection (*p* > 0.05). A statistically significant association was found between the treatment line and infection risk (*p* = 0.011). Patients undergoing second-line cranioplasty had an increased risk of infection compared to those treated in the first line (OR = 1.94, 95% CI: 1.16–3.26). Implant location was also significantly associated with infection occurrence (*p* = 0.010), with bifrontal cranioplasties carrying a higher risk compared to unilateral fronto-parieto-temporal implants (OR = 2.51, 95% CI: 1.22–5.19) (see Supplemental Table S3).

### 3.3. Infection Characteristics and Treatment Outcomes

Table 3 summarizes infection-related explants after PHA cranioplasty. Among the 76 patients with post-cranioplasty infection, 59 (77.6%) underwent implant removal. Infections were predominantly superficial (92.1%, n = 70), with a higher explantation rate in deep infections (100%, 6/6) compared to superficial ones (75.7%, 53/70).

While explantation occurred in all cases of deep infection (6/6), statistical analysis did not reveal a significant association between infection depth and explantation risk (Fisher’s exact test, *p* = 0.328). This is likely due to the low number of deep infections and the presence of a zero-count cell in the non-explant group, which limits statistical power. Although this result does not reach significance, the clinical observation remains relevant and warrants further investigation in larger cohorts.

Most infections were classified as early onset, occurring in 61.9% of cases (n = 47), with a higher likelihood of explantation (80.5%) than in late-onset infections (72.4%, 21/29).

In terms of infection management, 18 patients (23.7%) were treated with systemic antibiotics alone as an initial therapeutic approach. Among these, two patients (11.1%) required subsequent explantation. To explore the role of antibiotic duration, we pre-specified a binary variable to classify systemic antibiotic treatment as short (<4 weeks, n = 2) or prolonged (≥4 weeks, n = 16), based on commonly used clinical thresholds for bone and implant-associated infections. Notably, all patients who successfully resolved the infection without explantation had received broad-spectrum antibiotic combinations with activity against a wide range of bacteria, administered over an extended period (see Supplemental Table S2 for details on the specific agents used). Although the number of cases is limited, this categorical analysis suggests that short-course antibiotic therapy may be insufficient to control infection, particularly in the absence of surgical revision. Due to the small sample size and the presence of a zero-cell count, statistical inference is limited, and results should be interpreted cautiously.

Surgical revision without antibiotic treatment was performed in the 58 other patients (76.3%): 6 patients (7.9%) underwent explantation without reimplantation (i.e., implant removal without placement of a new prosthesis), 31 (40.8%) received a new implant made of different material, 16 (21%) received a backup PHA implant, 4 (5.3%) were reimplanted with a newly manufactured PHA device, and 1 (1.3%) was reimplanted with the same PHA implant.

Surgical debridement was reported in 11.8% of cases (n = 9), one of whom ultimately required explantation. Local (in situ) antibiotic therapy (see Supplemental Table S2) was administered in 13 patients (17.1%), 12 of whom also received concurrent systemic antibiotics. Among these 13, three patients (23.1%) required explantation: one who had not received systemic therapy and two who had received it for a limited duration. No explants occurred among patients treated with both in situ and adequately prolonged systemic antibiotic therapy.

Among the variables analyzed, infection management strategies, surgical debridement, and the use of in situ antibiotic therapy were significantly associated with implant explantation. Specifically, patients treated with systemic antibiotics alone showed a significantly lower risk of explantation compared to those undergoing surgical reoperation (OR = 0.0022, 95% CI: 0.0002–0.0258, *p* < 0.001). Similarly, surgical debridement was associated with a markedly reduced likelihood of implant removal (OR = 0.0197, 95% CI: 0.0022–0.1772, *p* < 0.001). The application of in situ antibiotic therapy was also significantly correlated with lower explantation rates (OR = 0.0375, 95% CI: 0.0083–0.1699, *p* < 0.001) (see Supplemental Table S4). These findings emphasize the importance of conservative treatment approaches, where feasible, in reducing the need for implant removal following post-cranioplasty infections. Given the small sample size of some subgroups and the presence of sparse data, the reported OR should be interpreted with caution. These findings are exploratory and hypothesis-generating, and further validation in larger cohorts is warranted.

### 3.4. Microbiological Findings

Microbiological analyses were conducted in most cases of infection-related explantation (Table 4).

Wound swabs were performed in 62 patients (81.6%), and pathogens were identified in all cases. Staphylococcus species were the most frequently isolated (n = 43, 69.3%), followed by non-staphylococcal bacteria (n = 19, 30.7%). Blood cultures were performed in 73 cases (96.1%) and showed similar distributions, with *Staphylococcus* spp. isolated in 52 patients (71.2%) and non-staphylococcal pathogens in 21 (28.8%). Where clinical data were available, the most common signs of infection included fever—either alone or in combination with other symptoms such as skin redness, pain, headache, seizures (in one case), radiological abnormalities, or surgical site dehiscence with implant exposure. Notably, in 38% of cases reporting clinical signs of infection, purulent discharge was also documented.

No statistically significant association was found between the type of pathogen isolated (*Staphylococcus* spp. vs. non-Staphylococcus) and the risk of explantation, either in wound swabs (*p* = 0.795) or in blood cultures (*p* = 0.497) (see Supplemental Table S4).

## 4. Discussion

Postoperative infection remains one of the most challenging complications following cranioplasty [5,6,7], often requiring implant removal and reoperation [16] and potentially impacting neurological recovery, aesthetic outcomes, and overall quality of life. This retrospective multicentric European study, encompassing 984 patients who underwent cranioplasty with custom-made porous hydroxyapatite (PHA) implants, provides one of the largest contemporary cohorts focused specifically on infection management in this setting, even if limited to a single heterologous material. Unlike our previous publication, which focused primarily on infection rates in adult and pediatric populations [12], this expanded analysis investigates the characteristics of infection, management strategies, and explantation risk, incorporating newly defined treatment categories and comparisons. Our findings offer valuable insights into infection characteristics, microbiology, infection management strategies, and outcomes, and contribute to refining current clinical approaches.

When comparing the whole population (984 cases) with the subset of patients who developed postoperative infections (76 cases, 7.7%), the only statistically significant factors associated with a higher risk of infection were the treatment line (first or second implant) and prosthesis location. These factors confirm previous findings, both concerning treatment line—with more complications after a second implant [17,18]—and regarding a higher risk of infections in bifrontal craniectomies with different materials [19].

The observed overall infection rate of 7.7% is slightly higher than previously reported for cranioplasty procedures [20,21], though this may be explained by the longer median follow up in our study [18,22]. It remains lower or comparable to infection rates reported with other heterologous materials [4,19,23,24]. The porous nature of PHA, while promoting osteointegration, may theoretically increase the risk of bacterial colonization. Nevertheless, osteointegration is often viewed as a key advantage of PHA in reconstructive neurosurgery, especially in complex or high-risk patients [18].

Most infections were superficial and of early onset (≤2 months post-cranioplasty), with *Staphylococcus* spp.—especially coagulase-negative staphylococci—being the predominant pathogens identified. This aligns with typical skin flora contamination pathways, suggesting perioperative or early postoperative contamination as the most likely source [25]. Notably, deep infections and late-onset infections, though less common, were consistently associated with a higher risk of explantation, underscoring the importance of early recognition and aggressive management [9].

The explantation rate among infected patients was high (77.6%), particularly in deep (100%) and early-onset infections (80.5%). However, our data support the hypothesis that conservative treatment with prolonged systemic antibiotic therapy—especially when combined with local antibiotic application—may obviate the need for explantation in selected superficial infections, as already published in a few case reports [9]. Indeed, among patients treated exclusively with antibiotics, those who received adequately prolonged, broad-spectrum regimens without surgical intervention exhibited high rates of infection resolution and implant retention. This finding highlights the potential value of a stratified, pathogen-targeted treatment algorithm, taking into account infection depth, timing, clinical severity, and patient factors. However, these findings should be interpreted with caution. Given the retrospective nature of the study and the clinician-driven choice of treatment, the observed association between antibiotic-only therapy and implant retention may reflect confounding by indication. Variables such as infection severity, depth, and timing—as well as differences in center-specific protocols—likely influenced treatment decisions. Therefore, the observed protective effect of conservative therapy should be considered hypothesis-generating and warrants further evaluation in prospective, controlled studies. Considering the limited sample size and sparse distribution of key outcome categories, we attempted a binary logistic regression model, but the results were unstable and not statistically reliable. For this reason, we opted to present only descriptive and bivariate analyses, acknowledging the exploratory nature of the findings.

Reimplantation strategies following explantation varied widely, with roughly two-thirds of patients receiving a new implant—either a different material (most frequently titanium or PEEK) or a newly manufactured PHA prosthesis. In a subset of cases, use of a backup PHA implant was pursued in the same surgery. While the latter remains exceptional, these approaches may be viable in selected low-virulence, superficial infections where complete eradication of the pathogen can be reasonably ensured. Such individualized management strategies require careful clinical judgment and should be supported by microbiological and imaging evidence.

The microbiological data confirmed the predominance of Gram-positive cocci, with *Staphylococcus* spp. accounting for approximately 70% of isolates. This reinforces the need for empiric antibiotic protocols to include agents effective against these organisms while awaiting culture results. The systematic use of both wound swabs and blood cultures in most cases allowed for precise pathogen identification and informed antibiotic tailoring, which is crucial for successful infection control and prevention of recurrence.

Interestingly, blood cultures were positive in nearly all patients in whom they were performed (96.1%), which is higher than typically reported in cranioplasty infections. This may reflect selection bias, as blood cultures were likely reserved for patients with overt clinical symptoms such as fever or systemic signs of infection. Additionally, some cases classified as superficial may have had undetected deeper involvement. In neurosurgical infections, blood cultures generally yield lower positivity rates—often below 60%—with even lower sensitivity in superficial infections [26]. These findings highlight the need for standardized protocols to guide when blood cultures should be collected in the context of suspected cranioplasty infection. For future studies, we recommend the implementation of a standardized protocol for culture collection.

It is also to be noted that all patients with clear clinical symptoms were explanted with no indication at all of tentative orthosis salvage.

This study has several strengths, including its large sample size, multicentric design, and standardized data collection within the framework of post-market clinical surveillance. However, some limitations must be acknowledged. The retrospective nature of the study may introduce selection bias, particularly in the reporting and classification of infection severity and outcomes. Additionally, variations in institutional practices and antibiotic treatment protocols may have influenced management strategies and outcomes. Finally, although PHA is widely used across Europe, the findings may not be fully generalizable to centers using different implant materials or surgical workflows.

## 5. Conclusions

Despite these limitations, our data support a tailored approach to infection management following cranial reconstruction. In particular, infections that are superficial, of early onset (≤2 months), and without systemic signs may often be successfully managed with prolonged antibiotic therapy, especially when systemic and local treatments are combined, avoiding explantation and secondary reconstruction. Conversely, deep infections and/or those with delayed onset or systemic involvement more frequently necessitate implant explantation. These findings suggest a pragmatic clinical pathway based on infection depth, timing, and severity, which may help guide decision-making in real-world settings. Future prospective studies and the development of standardized treatment protocols are warranted to validate this approach and optimize outcomes while preserving the regenerative benefits of PHA implants in cranial reconstruction.

## Figures and Tables

**Table 1:** Demographic and surgical characteristics of the study cohort.

	Number	%
**Patients**	984	100
**Sex**		
Female	351	35.7
Male	633	64.3
**Age**		
Median age	41.5	
Pediatric (2–13 years)	85	8.6
Female	32	37.7
Male	53	62.3
Adult (14+ years)	899	91.4
Female	319	35.5
Male	580	64.5
**Primary diagnosis**		
Trauma	562	57.1
Vascular disease	205	20.8
Tumor	171	17.4
Malformation	31	3.2
Other	15	1.5
**Line of treatment**		
First line	795	80.8
Second line	189	19.2
**Location**		
Fronto-parieto-temporal	858	87.2
Bifrontal	62	6.3
Other	64	6.5
**Complications**	128	13
Infection	76	7.7
Fracture	22	2.2
Displacement	8	0.8
Other	22	2.2

**Table 2:** Characteristics of post-cranioplasty infection group.

	Number	%
**Infection**	76	100
**Sex**		
Female	29	38.2
Male	47	61.8
**Age**		
Median age	42.5	
Pediatric (2–13 years)	9	11.8
Female	2	22.2
Male	7	77.8
Adult (14+ years)	67	88.2
Female	27	40.3
Male	40	59.7
**Primary diagnosis**		
Trauma	43	56.6
Vascular disease	16	21
Tumor	11	14.5
Malformation	4	5.3
Other *	2	2.6
**Line of treatment**		
First line	53	69.7
Second line	23	30.3
**Location**		
Fronto-parieto-temporal	61	80.3
Bifrontal	10	13.2
Other	5	6.6

* Other complications include scar retraction, fistula formation, hematoma, hemorrhage, and tumor relapse.

**Table 3:** Summary of infection-related explants (n = 59) after PHA cranioplasty.

	n (%)	Explant, n (%)
**Infection site**		
Deep	6 (7.9)	6 (100)
Superficial	70 (92.1)	53 (75.7)
**Infection onset**		
Early	47 (61.9)	38 (80.5)
Late	29 (38.2)	21 (72.4)
**Infection management**		
Antibiotic treatment only	18 (23.7)	2 (11.1)
Explantation without reimplantation	6 (7.9)	6 (100)
Reoperation with different implant material	31 (40.8)	31 (100)
Reoperation using backup PHA implant	16 (21)	16 (100)
Reoperation with new PHA implant	4 (5.3)	4 (100)
Reoperation with the same PHA implant	1 (1.3)	-
**Surgical debridement**		
Yes	9 (11.8)	1 (11.1)
No	66 (86.8)	57 (86.4)
ND	1 (1.3)	1 (100)
**Antibiotic treatment in situ**		
Yes	13 (17.1)	3 (23.1)
No	63 (82.9)	56 (88.9)

**Table 4:** Microbiological sampling and pathogen identification in cases of infection-related explants.

	n (%)	Explant, n (%)
**Wound swab**		
Yes	62 (81.6)	47 (75.8)
*Staphylococcus* spp.	43 (69.3)	33 (76.7)
Non-staphylococcal bacteria	19 (30.7)	14 (73.7)
No	7 (9.2)	5 (71.4)
ND	7 (9.2)	7 (100)
**Blood culture**		
Yes	73 (96.1)	56 (76.7)
*Staphylococcus* spp.	52 (71.2)	41 (78.8)
Non-staphylococcal bacteria	21 (28.8)	15 (71.4)
ND	3 (3.9)	3 (100)

## Data Availability

The data presented in this study are not publicly available due to patient privacy considerations and institutional regulations. A de-identified dataset and variable definitions can be made available upon reasonable request to the corresponding author.

## Supplementary Materials

The following supporting information can be downloaded at https://www.mdpi.com/article/10.3390/jcm14186443/s1. Text File S1: Data collection protocol; Table S1: Participating European centers and neurosurgeons; Table S2: Antibiotics summary table; Table S3: Univariate analysis of infection risk factors; Table S4: Univariate analysis of explantation risk factors.

## References

[1] Rossini Z., Franzini A., Zaed I., Zingaretti N., Nicolosi F., Zanotti B. (2020). Custom-Made Porous Hydroxyapatite Cranioplasty in Patients with Tumor Versus Traumatic Brain Injury: A Single-Center Case Series. World Neurosurg..

[2] Maricevich J.P.B.R., Cezar-Junior A.B., de Oliveira-Junior E.X., Morais e Silva J.A., da Silva J.V.L., Nunes A.A., Almeida N.S., Azevedo-Filho H.R.C. (2019). Functional and aesthetic evaluation after cranial reconstruction with polymethyl methacrylate prostheses using low-cost 3D printing templates in patients with cranial defects secondary to decompressive craniectomies: A prospective study. Surg. Neurol. Int..

[3] Ganau M., Cebula H., Fricia M., Zaed I., Todeschi J., Scibilia A., Gallinaro P., Coca A., Chaussemy D., Ollivier I. (2020). Surgical preference regarding different materials for custom-made allograft cranioplasty in patients with calvarial defects: Results from an internal audit covering the last 20 years. J. Clin. Neurosci..

[4] Henry J., Amoo M., Taylor J., O’Brien D.P. (2021). Complications of Cranioplasty in Relation to Material: Systematic Review, Network Meta-Analysis and Meta-Regression. Neurosurgery.

[5] Ashraf M., Choudhary N., Kamboh U.A., Raza M.A., Sultan K.A., Ghulam N., Hussain S.S., Ashraf N. (2022). Early experience with patient-specific low-cost 3D-printed polymethylmethacrylate cranioplasty implants in a lower-middle-income-country: Technical note and economic analysis. Surg. Neurol. Int..

[6] Policicchio D., Casu G., Dipellegrini G., Doda A., Muggianu G., Boccaletti R. (2020). Comparison of two different titanium cranioplasty methods: Custom-made titanium prostheses versus precurved titanium mesh. Surg. Neurol. Int..

[7] Celik H., Kurtulus A., Yildirim M.E., Tekiner A., Erdem Y., Kantarci K., Kul H., Bayar M.A. (2022). The Comparison of Autologous Bone, Methyl-Methacrylate, Porous Polyethylene, and Titanium Mesh in Cranioplasty. Turk. Neurosurg..

[8] Kim M.J., Lee H.B., Ha S.K., Lim D.J., Kim S.D. (2021). Predictive Factors of Surgical Site Infection Following Cranioplasty: A Study Including 3D Printed Implants. Front. Neurol..

[9] Di Rienzo A., Colasanti R., Dobran M., Formica F., Della Costanza M., Carrassi E., Aiudi D., Iacoangeli M. (2022). Management of infected hydroxyapatite cranioplasty: Is salvage feasible?. Brain Spine.

[10] Yeap M.C., Tu P.H., Liu Z.H., Hsieh P.C., Liu Y.T., Lee C.Y., Lai H.Y., Chen C.T., Huang Y.C., Wei K.C. (2019). Long-Term Complications of Cranioplasty Using Stored Autologous Bone Graft, Three-Dimensional Polymethyl Methacrylate, or Titanium Mesh After Decompressive Craniectomy: A Single-Center Experience After 596 Procedures. World Neurosurg..

[11] Bečulić H., Spahić D., Begagić E., Pugonja R., Skomorac R., Jusić A., Selimović E., Mašović A., Pojskić M. (2023). Breaking Barriers in Cranioplasty: 3D Printing in Low and Middle-Income Settings—Insights from Zenica, Bosnia and Herzegovina. Medicina.

[12] Mannella F.C., Faedo F., Fumagalli M., Norata G.D., Zaed I., Servadei F. (2024). Long-Term Follow-Up of Custom-Made Porous Hydroxyapatite Cranioplasties: Analysis of Infections in Adult and Pediatric Patients. J. Clin. Med..

[13] Horan T.C., Gaynes R.P., Martone W.J., Jarvis W.R., Emori T.G. (1992). CDC definitions of nosocomial surgical site infections, 1992: A modification of CDC definitions of surgical wound infections. Am. J. Infect. Control.

[14] Mangram A.J., Horan T.C., Pearson M.L., Silver L.C., Jarvis W.R. (1999). Guideline for Prevention of Surgical Site Infection, 1999. Centers for Disease Control and Prevention (CDC) Hospital Infection Control Practices Advisory Committee. Am. J. Infect. Control.

[15] CDC (2023). National Healthcare Safety Network (NHSN) Surgical Site Infection (SSI) Event. https://www.cdc.gov/nhsn/pdfs/pscmanual/9pscssicurrent.pdf.

[16] Oliver J.D., Banuelos J., Abu-Ghname A., Vyas K.S., Sharaf B. (2019). Alloplastic Cranioplasty Reconstruction: A Systematic Review Comparing Outcomes With Titanium Mesh, Polymethyl Methacrylate, Polyether Ether Ketone, and Norian Implants in 3591 Adult Patients. Ann. Plast. Surg..

[17] Chen K., Liang W., Zhu Q., Shen H., Yang Y., Li Y., Li H., Wang Y., Qian R. (2023). Clinical Outcomes After Cranioplasty With Titanium Mesh, Polyetheretherketone, or Composite Bone Cement: A Retrospective Study. J. Craniofac. Surg..

[18] Fricia M., Nicolosi F., Ganau M., Cebula H., Todeschi J., des Neiges Santin M., Nannavecchia B., Morselli C., Chibbaro S. (2019). Cranioplasty with Porous Hydroxyapatite Custom-Made Bone Flap: Results from a Multicenter Study Enrolling 149 Patients Over 15 Years. World Neurosurg..

[19] Wesp D., Krenzlin H., Jankovic D., Ottenhausen M., Jägersberg M., Ringel F., Keric N. (2022). Analysis of PMMA versus CaP titanium-enhanced implants for cranioplasty after decompressive craniectomy: A retrospective observational cohort study. Neurosurg. Rev..

[20] Amendola F., Vaienti L., Carbonaro R., Nataloni A., Barbanera A., Zingaretti N., Pier C.P., Zanotti B. (2022). The Antibiotic Immersion of Custom-Made Porous Hydroxyapatite Cranioplasty: A Multicentric Cohort Study. J. Craniofac. Surg..

[21] Messina R., Speranzon L., de Gennaro L., Nigri E.M., Dibenedetto M., Bozzi M.T., Delvecchio C., Signorelli F. (2024). Early osteointegration in “one-step” resection and reconstruction using porous hydroxyapatite custom implants for skull-infiltrating tumors: A monocentric prospective series. Acta Neurochir..

[22] Pfnür A., Tosin D., Petkov M., Sharon O., Mayer B., Wirtz C.R., Knoll A., Pala A. (2024). Exploring complications following cranioplasty after decompressive hemicraniectomy: A retrospective bicenter assessment of autologous, PMMA and CAD implants. Neurosurg. Rev..

[23] Bader E.R., Kobets A.J., Ammar A., Goodrich J.T. (2022). Factors predicting complications following cranioplasty. J. Craniomaxillofac. Surg..

[24] Ebel F., Schön S., Sharma N., Guzman R., Mariani L., Thieringer F.M., Soleman J. (2023). Clinical and patient-reported outcome after patient-specific 3D printer-assisted cranioplasty. Neurosurg. Rev..

[25] Zaed I., Iaccarino C., Faedo F., Grillini L., Galassi E., Dotti A., Nataloni A., Mannella F.C., Cardia A. (2025). Cranioplasty infection in porous hydroxyapatite: Potential antibacterial properties. J. Appl. Biomater. Funct. Mater..

[26] Carone G., Bonada M., Belotti E.G., D’Angeli E., Piccardi A., Doniselli F.M., Gubertini G., Casali C., DiMeco F., Del Bene M. (2025). Post-craniotomy infections: A point-by-point approach. Brain Spine.

[27] Luo X., Tham Y.C., Giuffrè M., Ranisch R., Daher M., Lam K., Eriksen A.V., Hsu C.W., Ozaki A., Moraes F.Y. Reporting guideline for the use of Generative Artificial intelligence tools in MEdical Research: The GAMER Statement. BMJ Evid. Based Med..

