# Supplementary material for: A Detailed Study of Infection Following Custom-Made Porous Hydroxyapatite Cranioplasty: Risk Factors and How to Possibly Avoid Device Explantation
Source: J Clin Med. 2025 Sep 12;14(18):6443. doi: 10.3390/jcm14186443 (PMC12471258; doi:10.3390/jcm14186443)
Supplement: Supplementary file 1 [file jcm-14-06443-s001.zip › jcm-3831626-supplementary.pdf]

Supplemental Table S1. Participating European centers and neurosurgeons

Country	City	Hospital	Neurosurgeons
Belgium	Gent	Ghent University Hospital	G. Hallaert
France	Lyon	Hospices Civil de Lyon	C. Mottolese
	Paris	Hôpital Lariboisière	S. Chibbaro
	Paris	Centre hospitalier Sainte-Anne	J. Pallud
	Strasbourg	Hôpital de Hautepierre	S. Chibbaro
	Tours	Hôpital Bretonneau	P. Francois
Ireland	Dublin	Beaumont Hospital	D. Crimmins
		Temple Street children's hospital	D. Crimmins
Italy	Alessandria	Ospedale SS. Antonio e Biagio e Cesare Arrigo	A. Barbanera
	Ancona	Ospedali Riuniti	A. Di Rienzo
			M. Luzi
	Aosta	Ospedale Regionale della Valle D'Aosta	A. Barbanera
	Bari	Policlinico di Bari – Ospedale Giovanni XXIII	F. Signorelli
	Bologna	Ospedale Bellaria	E. Serchi
		Ospedale Maggiore	E. Serchi
	Brescia	Spedali Riuniti	R. Stefini
			C. Cereda
	Catania	Ospedale Cannizzaro	M. Fricia
	Gemona del Friuli	Ospedale San Michele	P.C. Parodi
	Genova	Ospedale Gaslini	M. Pavanello
	Legnano	ASST Ovest Milanese	R. Stefini
	Mantova	Ospedale Poma	B. Zanotti
	Napoli	Ospedale Santobono	P. Spennato
			C. Ruggiero
	Padova	Ospedale Civile	R. Faggin,
	Palermo	Policlinico Universitario	F. Graziano
	Parma	Ospedale Maggiore	C. Iaccarino
	Perugia	Ospedale Santa Maria della Misericordia	G. Ghetti
	Roma	Bambino Gesù Children's Hospital	P. Palma
	Rozzano	Humanitas Research Hospital	F. Servadei
	Udine	Ospedale Santa Maria della Misericordia	B. Zanotti
	Vicenza	Ospedale San Bortolo	G.P. Zambon
Switzerland	Lausanne	Centre Hospitalier Universitaire Vaudois	R. Maduri

Supplemental Table S2. Antibiotics summary table

Therapy approach	Treatment duration	Antibiotic class	Antibiotic molecules	Antimicrobial spectrum
Systemic	2 weeks	Rifamycin + Glycylcycline + Polymyxin	Rifampicin + Tigecycline + Colistin	Gram+, Gram−, anaerobes, MDR organisms
	4 weeks	Cephalosporins (3rd gen.) + Nitroimidazoles + Aminoglycosides	Cefotaxime + Metronidazole + Gentamicin	Gram+, Gram−, anaerobes
	12 weeks	Carbapenems + Oxazolidinones	Meropenem + Linezolid	Gram+, Gram−, MDR organisms, MRSA
	12 weeks	Glycopeptides + Aminoglycosides + Cephalosporins (3rd/4th gen.)	Teicoplanin + Gentamicin + Ceftazidime	Gram+ (also MRSA), Gram− (also Pseudomonas spp.)
	8 weeks	Glycopeptides + Carbapenems	Vancomycin + Meropenem	Gram+ (also MRSA), Gram−, anaerobes
	4 weeks	Fluoroquinolones + Rifamycin	Levofloxacin + Rifampicin	Gram+, Gram−, intracellular bacteria
	4 weeks	Fluoroquinolones + Glycopeptides	Levofloxacin + Teicoplanin	Gram+, Gram−
In situ	ND	Rifamycin + Glycylcycline + Polymyxin	Rifampicin + Tigecycline + Colistin	Gram+, Gram−, anaerobes, MDR organisms
	ND	Carbapenems	Meropenem	Gram+, Gram−, anaerobes
	ND	Glycopeptides	Vancomycin	Gram+ (also MRSA)
	ND	Glycopeptides + Carbapenems	Vancomycin + Imipenem/Cilastatin	Gram+ (also MRSA), Gram−, anaerobes, MDR organisms
	ND	Fluoroquinolones + Rifamycin	Levofloxacin + Rifampicin	Gram+, Gram−, intracellular bacteria
	ND	Fluoroquinolones + Glycopeptides	Levofloxacin + Teicoplanin	Gram+, Gram−
	ND	Oxazolidinones	Linezolid	Gram+ (also MRSA and VRE)

ND = Not Defined; MRSA = Methicillin-resistant Staphylococcus aureus; MDR organisms = Multidrug-resistant organisms, including bacteria resistant to three or more classes of antibiotics; VRE = Vancomycin-resistant Enterococcus.

Supplemental Table S3. Univariate analysis of infection risk factors

Variable	p-value	Odds Ratio (OR)	95% CI
Sex (M vs F)	0.637	0.89	0.55–1.44
Age group (Pediatric vs Adult)	0.301	0.68	0.33–1.42
Primary diagnosis	0.693	-	-
Cranioplasty treatment line (2nd vs 1st)	0.011	1.94	1.16–3.26
Implant location (Bifrontal vs FPT)	0.01	2.51	1.22–5.19

Supplemental Table S4. Univariate analysis of explantation risk factors

Variable	p-value	Odds Ratio (OR)	95% CI
Infection management strategy (antibiotics only vs others)	<0.001	0.02	0.00–0.09
Surgical debridement (Yes vs No)	<0.001	0.11	0.01–0.91
In situ antibiotics (Yes vs No)	<0.001	0.20	0.04–0.95
Wound swab (Staphylococcus vs non-Staphylococcus)	0.795	1.18	0.34–4.11
Blood culture (Staphylococcus vs non-Staphylococcus)	0.497	1.46	0.43–4.96
